# How is energy poverty linked with citizen perceptions of financial support for low-carbon housing?

**DOI:** 10.1007/s13280-026-02364-4

**Published:** 2026-03-17

**Authors:** Enni Ruokamo, Santtu Karhinen, Teemu Kemppainen, Jouni Räihä, Anna Strandell, Juhani Marttila, Liina Häyrinen, Anne Toppinen

**Affiliations:** 1 Finnish Environment Institute (Syke), Latokartanonkaari 11, 00790 Helsinki, Finland; 2 Department of Geosciences, University of Helsinki, P.O. Box 4, 00014 Helsinki, Finland; 3 Department of Chemistry and Sustainable Technology, University of Eastern Finland, P.O. Box 111, 80101 Joensuu, Finland; 4 Natural Resources Institute Finland, Latokartanonkaari 9, 00790 Helsinki, Finland; 5 Department of Forest Sciences, University of Helsinki, Latokartanonkaari 7, 00014 Helsinki, Finland; 6 Jyväskylä University School of Business and Economics, University of Jyväskylä, P.O. Box 35, 40014 Jyväskylä, Finland

**Keywords:** Attitude, Energy poverty, Household, Subsidy, Survey

## Abstract

Housing affordability and climate sustainability are increasingly interlinked in Europe, as housing costs strain household budgets and the residential sector contributes significantly to greenhouse gas emissions. This study investigates Finnish public perceptions of decarbonization housing policies, particularly views on financial support for the transition to low-carbon housing. Using nationally representative survey data from 2022, combined with housing registry and price data, we analyze how both objective and subjective indicators of energy poverty are linked with support for public funding. The findings demonstrate strong public support, particularly among women, urban residents, and those using oil heating. Objective energy poverty correlates positively with policy support, and subjective economic well-being mediates this relationship. These findings contribute to the growing literature on housing, energy poverty, and climate policy acceptance, underscoring the importance of addressing social vulnerabilities to secure equitable and widely supported low-carbon housing transitions.

**Supplementary Information:**

The online version contains supplementary material available at 10.1007/s13280-026-02364-4.

## Introduction

A rising challenge throughout the world is the growth of social and spatial polarization, crucially connected to real and perceived affordability challenges of citizens. Typically, housing costs form a large part of total living expenses. In 2023, EU households allocated approximately 20% of their disposable income to housing costs (European commission 2024). At the same time, buildings represent about a third of energy-related greenhouse gas emissions in the EU (EEA 2024) that necessitates significant investments in energy renovations and energy efficiency technologies. Social housing policies are therefore crucially intertwined with climate policies (Bergquist et al. 2020), with citizens being the key societal group in terms of acceptability and legitimacy.

In this study, we use population-based survey data from Finland to examine citizen perceptions of low-carbon housing policies. More concretely, we study whether citizens consider that society should financially support their transition to low-carbon housing. Connected to this, there has been some research interest on consumer attitudes and acceptance toward specific measures or policies that are associated to low-carbon transition in housing (see Section “Energy Poverty and citizen perceptions of policies”).

The possibility to attain low-carbon housing is connected to prices and affordability, but also other systemic matters. Dubois et al. (2019) find that household living situations (demographics, size of home) greatly influence the household potential to reduce their footprint, even more than country or city location. Thus, policies could attempt to address these barriers. Currently, the EU guidance for Energy Performance of Buildings Directive (Directive 2024/1275) positions that housing policies should be crafted with the vulnerable groups as priority.

Energy poverty is a concept of vulnerability connected to the ability to maintain good living condition in your home. Objective energy poverty refers to the measurable ability of a household to secure adequate energy services, such as heating and cooling, based on quantifiable indicators like energy costs and income. In addition to objective energy poverty indicators, feeling subjectively energy poor is found to be common and to capture to some extent different part of the population (Agbim et al. 2020; Maier and Dreoni 2026).

To our knowledge, research examining the impact of energy poverty on attitudes toward climate and energy policies remains extremely limited (Chapman and Okushima 2019). Little is known regarding how energy poverty is reflected in citizen views of low-carbon housing policy. Thus, special focus in our analysis is given to following research questions:

I. Are data-based objective energy poverty indicators associated with the perceptions of financial support for low-carbon housing?
II. Is this potential association of objective energy poverty with the policy support mediated via subjective economic well-being?

Tested objective energy poverty indicators are: (i) estimated housing-related energy costs divided by household income (i.e., energy burden) and (ii) over 10% of household income is spent on housing-related energy costs. Subjective economic well-being is measured via response to a survey question “How well do you think your household is managing financially today?” Interlinkages between the policy perception and demographics are studied as well. These insights are vital, as, according to Kortetmäki and Järvelä (2021), neglecting the vulnerability impacts of climate policies creates a risk of an increase in social segregation and feeling of injustice further negatively impacting the welfare among people.

To study connections between energy poverty, demographics, and perceptions of climate-wise housing policy, survey data collected from the Finnish citizens in 2022 as well as building registry and energy price data are utilized. Then, an ordered logit regression is used to analyze the linkage between dependent and independent variables.

Finland provides an interesting context for this study since it has set ambitious goals to reach national-level carbon neutrality by year 2035, 15 years before than the EU target year. In this cold northern country with high seasonal heating costs, 17.4% of disposable income was spent on housing expenses in 2023 (Official Statistics of Finland 2025). There have been several public support schemes that provide grants for deep energy retrofits and for replacing oil boilers with heat pump systems, plus enhanced tax credits. From the year 2026, every new residential flat must be accompanied by carbon footprint calculation accounting whole-life carbon footprint, steering new housing further toward intrinsically low carbon (Building Act 2023). Taken together the geographic context, importance of housing sector in household income structure, and range of relevant policy measures targeting socially equitable low-carbon housing, the choice of target country is well justified.

### Energy poverty and indicators

Oxford dictionary defines energy poverty as inability to attain sufficient energy services, whereas the European Commission (2025) states it occurs when energy consumption needs to be reduced to a degree that it impacts health and well-being. Research interest in the topic has gained considerable traction in recent years following the seminal works of Bradshaw and Hutton (1983) and Boardman (1991). Examining prevalence of energy poverty or energy vulnerability has evolved from strictly fuel-related measure looking chiefly electricity, heating or cooking fuel access (Moore 2012) to a concept that even more widely encompasses the complex dynamics of energy use in households (Kajoskoski et al. 2025).

Energy poverty is known to be associated with various demographics with better consensus for positive association with higher age, lower education, larger family size, lower income, rural and colder areas, and detached house dwelling (del Río et al. 2025). There are numerous indices that attempt to catch people in or at risk of falling into energy poverty (Herrero 2017; Deller et al. 2021; Siksnelyte-Butkiene et al. 2021; Croon et al. 2023; Menyhért 2024). A widely used subjective measure, origins of which can be tracked to Lewis (1982), follows the household inability to keep home sufficiently warm (or increasingly cool) and is typically subjectively measured via surveys due to data limitations. Other measures address affordability and objectivity by considering costs and income in calculating the so-called energy burden. In energy cost–income ratio, the most common derived objective energy poverty indicator is the “10% rule” introduced by Boardman (1991), which evaluates the share of households spending more than 10% of income on energy costs. Further research has increasingly introduced absolute or relative income-level measures into the equation to enable to better track the truly struggling households, such as households who spend more than twice national median (2M) and households who spend below half of the national median (M/2) to energy costs (Kajoskoski et al. 2025).

Objective energy poverty indicators offer a straightforward means of assessing the prevalence of energy poverty. However, they can fall short in capturing inadequate access to energy services and behavioral and contextual differences, and suffer from measurement and data limitations. Thus, subjective energy poverty assessments may better reflect contexts that are not fully captured by objective indicators. A typical subjective assessment question is already noted “can your household afford to keeping the home adequately warm,” and another example is “do you find your household’s energy expenditures a significant burden, considering your household’s income” (Numminen et al. 2024). Indeed, existing research shows that objective and subjective indicators of energy poverty often yield divergent results (Numminen et al. 2024; Maier and Dreoni 2026).

Globally energy poverty is recognized as a massive problem with over a billion people suffering from it according to Min et al. (2024). In the European union, Eurostat (2025) monitors the household inability to keep home adequately warm, a measure which can be viewed from the energy poverty hub. Following the energy crisis, the EU 27 average rebounded to 9.3% in 2022 and has maintained close to 10% of EU households ever since.

Maier and Dreoni (2026) examine energy poverty with different indicators across the European Union. They note that different indicators give vastly different results with very often little overlap in between them with expenditure-based indicators 2M and M/2 implicating around 17% energy poverty rate in the EU, roughly two times more than the subjective indicators used in the study. Both Maier and Dreoni (2026) and Numminen et al. (2024) note that the situation in Finland as a northern country looks the worst with the median income indicators estimating that roughly one-third of Finns would be energy poor under M/2 indicator, although it may reflect preferences and investments rather than deprivation, and nearly 20% with the 2M indicator in 2015, with more than half of the citizens classifiable as energy poor under some of the measures examined. Using Finnish survey data from detached homeowners collected in 2022, Numminen et al. (2024) estimate an energy poverty rate of 18% based on the 10% rule indicator.

### Energy poverty and citizen perceptions of policies

Previous research has extensively examined consumer attitudes toward and acceptance of measures and policies supporting the low-carbon transition in the housing sector. This literature covers a wide range of solutions, including the adoption of solar panels (Faiers and Neame 2006; Tham et al. 2025), the use of wood as a building material (Kylkilahti et al. 2020; Ruokamo et al. 2024a), nature-based solutions (Anderson and Renaud 2021), and the uptake of low-carbon buildings (Alam et al. 2020). Some studies adopt a broader perspective by examining multiple measures and policies simultaneously. For example, Rhodes et al. (2017) and Odland et al. (2023) examine public support for several market-based, regulatory, and voluntary climate policies using survey data collected from Canadian citizens. Similarly, Schmidt et al. (2024) investigate public acceptance of carbon pricing and other housing-related policies in Germany, while Douenne and Fabre (2020) conduct a comparable analysis for France. Spandagos et al. (2022) analyze support for renewable energy policies as well as adoption decisions related to individual renovation actions across the EU, while Peñasco (2024) examines household preferences for energy efficiency policies in the UK and their relationship to actual energy efficiency investments. Overall, this body of research suggests that support for low-carbon transition policies and actions is shaped by a range of factors, including sociodemographic characteristics, individual attitudes, and broader sociopolitical dimensions.

Evidence on how energy poverty shapes attitudes toward climate and energy policies remains very limited. Chapman and Okushima (2019) report that the energy-poor households had a negative attitude toward the low-carbon energy transition in Japan. Irwin (2025) notes that climate actions and social well-being must be addressed together as deeply interconnected challenges, with inclusive policies that reflect the realities of marginalized groups. While direct research linking energy poverty to policy attitudes is scarce, a larger body of literature examines the relationship between energy poverty and energy efficiency actions. Drawing on a literature review primarily focused on the UK and other European countries, Tozer et al. (2023) highlight that for households vulnerable to energy poverty, the main motivating factors for undertaking energy retrofits were financial savings, perceived improvements in health and quality of life, and a sense of social inclusion. Kosmopolous et al. (2020) report that in Greece, energy poverty does not necessarily diminish environmental awareness but significantly limits households’ willingness to invest in renewable energy solutions unless these offer clear financial benefits.

Generally, existing literature has examined both the conceptualization and measurement of energy poverty as well as its prevalence across regions (see Section “Energy poverty and indicators”). However, research addressing the relationship between energy poverty and citizens’ perceptions of low-carbon housing policies remains scarce, with current literature concentrating on households’ adoption of specific low-carbon actions rather than broader policy attitudes. As a result, a clear gap persists in understanding the interplay between objectively measured energy poverty and subjective assessments of economic well-being, as well as their influence on attitudes toward the low-carbon transition. This study addresses this gap by calculating objective energy poverty indicators, namely the energy cost–income ratio (i.e., energy burden) and the 10% rule, and empirically linking them together with subjective feelings of economic deprivation, to household perceptions of financial support for low-carbon housing.

## Materials and methods

### Data

This study uses data from a national survey examining Finnish citizens' preferences regarding climate-wise housing. The survey gathered information, for instance, on respondents’ attitudes toward climate-wise housing, general views of housing as well as housing and sociodemographic characteristics (see the survey questions used in this study in Supplementary Information, Appendix S1). The survey was developed iteratively through researcher workshops and piloted in two rounds. It was conducted in Finnish, Swedish, and English language in spring of 2022 using a random sample of 10 000 individuals drawn from the Finnish population information system. A total of 1448 responses were collected (14.5% response rate). While respondents were slightly older, more educated, and included more women than the general population, the sample was broadly representative in terms of household size, region, and housing type. More details about survey development and representativeness can be found from Ruokamo et al. (2024b). When constructing the energy burden variable, we used survey information on building type (detached house, row house, apartment building), dwelling floor area, year of construction, primary heating system, supplementary heating with an air-source heat pump, presence of a solar PV system, gross household income, and household size.

**Fig. 1:**
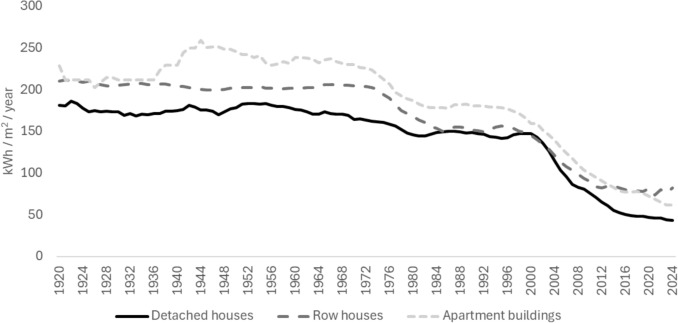
Specific consumption values by building type and construction year

Because the survey did not include information on the quantities or prices of energy consumed, we estimated them using several external data sources. First, we used Finland’s Energy Performance Certificate (EPC) registry to compile energy use intensities (kWh/m2), categorized by building type (detached houses, row houses, and apartment buildings) and year of construction. These intensities cover energy used for space heating and domestic hot water heating. By law, the EPC registry contains certificates only for buildings that are rented, sold, or newly constructed. Consequently, the estimated energy use intensities show considerable variability, especially for older buildings, because many of them have never been sold or rented and therefore do not appear in the registry. To address this, we applied a seven-year moving average (construction year ± 3 years) to smooth the time series and reduce excess variability (Fig. 1). We matched the estimated intensities to each survey dwelling by building type and construction year and calculated total annual energy demand by multiplying the intensity by the floor area reported in the questionnaire.

The estimated energy demand does not directly correspond to purchased energy, because the amount of purchased energy depends on the efficiency of the heating system. Therefore, we converted heat demand into purchased energy using heating method-specific typical efficiencies from the EKOREM-tok model (Mattinen et al. 2014): 98% for district heating, 95% for direct electric heating, 55% for wood fuel heating, and 80% for oil heating. For heat pumps, we assumed coefficients of performance (COP) of 3.0 for ground-source heat pumps, 2.5 for air-to-water heat pumps, and 2.0 for exhaust-air heat pumps. Supplementary heating systems and on-site solar energy production reported in the questionnaire were also incorporated into the estimates. If the dwelling included an air-source heat pump, we assumed a 33% reduction in the heat demand. If solar panels were present, we assumed a 10% reduction in total purchased energy.

In addition to space and water heating, we estimated typical other electricity use (kWh/m3/year), including electricity for household appliances, lighting, cooking, electric saunas, and other equipment, separately for each building type. The estimates were derived by combining Statistics Finland data on household energy consumption (Statistics Finland 2025a) with building stock data (Statistics Finland 2025b). The resulting values for other electricity use were 18.2, 18.5, and 16.9 kWh/m3/year for apartment buildings, row houses, and detached houses, respectively.

The survey responses were collected in 2022, a period marked by increased energy prices due to the war in Ukraine. Because the survey did not record households’ actually paid energy prices or energy supply contract types, prices were estimated using several statistical sources The average electricity price in the Finnish Energy Authority’s obligation-to-supply-type consumer price series in 2022 was 9.82 cents/kWh (Statistics Finland 2025c). Distribution charges vary by location, as electricity distribution is provided by local operators that function as regulated natural monopolies. We obtained typical uniform distribution tariffs from the Finnish Energy Authority (2025) and matched them to respondents’ locations. Respondents’ distribution charges for apartment buildings ranged from 6.5 to 19.3 cents/kWh, and from 4.8 to 9.9 cents/kWh for detached houses and row houses. Distribution charges include an electricity tax of 2.79 cents/kWh. The price of district heating also varies between municipalities. In the respondents' municipalities, in 2022 the price ranged from 5.7 to 12.1 cents/kWh (Finnish Energy 2025). The price of wood fuel was assumed to be 70 euros/m^3^ (loose volume) for mixed wood. Using an energy content of approximately 780 kWh/m^3^ at 20% moisture (Alakangas et al. 2016), this corresponds to 9.0 cents/kWh. The cost of oil was 16.8 cents/kWh (Statistics Finland 2025c). All prices include 24.0% value-added tax.

Finally, after estimating purchased energy for space and water heating, other household electricity use, and energy prices, we calculated total annual household energy costs. To formulate energy burden variable, we used household income information from the survey. Income was reported as gross income (including income transfers) and needed to be converted to net income by deducting taxes and taxlike payments. Because income was reported at the household level, we assumed it was distributed evenly among adult household members. The deductions include state income tax, municipal tax (average rate 20.01%), church tax (1.39%), the public broadcasting tax (2.50% of income exceeding 14 000€), health insurance contributions (health care 0.53% and daily allowance 1.18%), the employee’s earnings-related pension contribution (7.15%), and the unemployment insurance contribution (1.50%). In addition, standard automatic deductions applied by the Finnish Tax Administration were approximated, which lowers the effective tax rate. The gross-to-net conversion was calculated separately for each gross income bracket (from 10 000€ to 100 000€) to account for the progressive structure of income taxation and income-dependent contributions.

The estimated energy cost was then divided by household’s net income (variable *Energy burden*). If this ratio exceeded 10%, indicating that more than 10% of household income is spent on housing-related energy costs, the energy poverty indicator (variable *Energy poverty 10%)* was assigned a value of 1 and 0 otherwise.

### Empirical approach

**Fig. 2:**
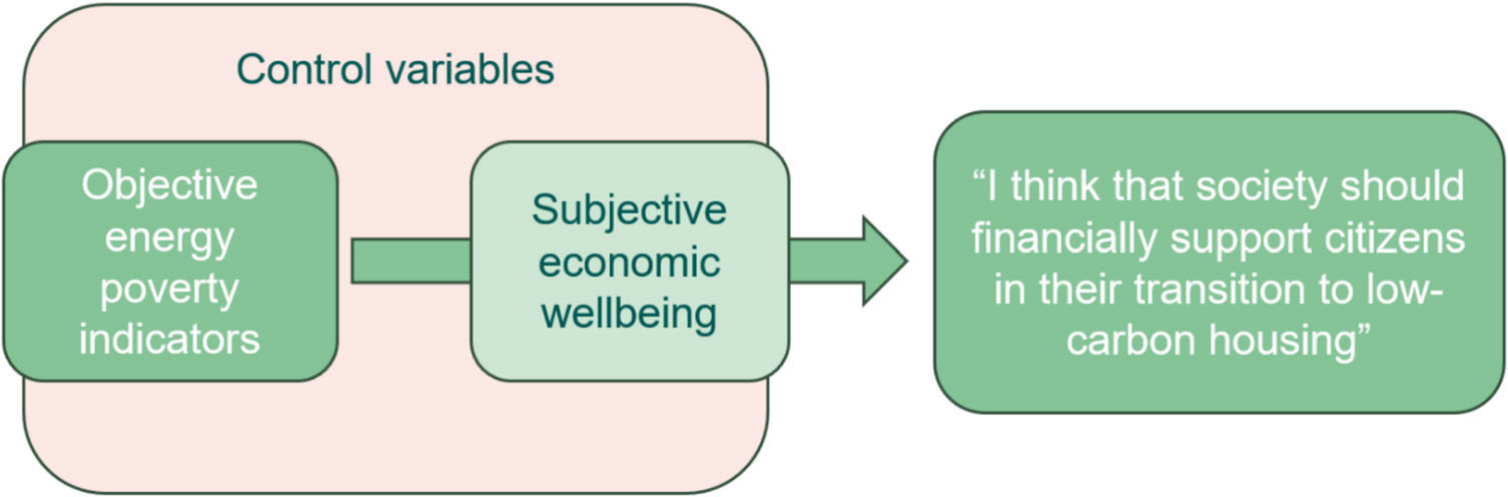
Empirical approach

The empirical approach of this study is shown in Fig. 2. The main hypotheses that we are interested in testing are as follows:

#### H1:

Objective energy poverty is associated with higher acceptance for financially supporting citizens in transition to low-carbon housing. Based on general rational choice framework, where an individual chooses the option that maximizes their own well-being (Becker 1976), it appears a priori logical to expect that individuals prefer policies that are useful or helpful in their life situation.

#### H2:

The association of objective energy poverty with higher acceptance for financially supporting citizens in transition to low-carbon housing runs partly via subjective economic well-being. The situation of energy poverty is experienced as a weakened state subjective economic well-being, which incentivizes the mentioned public policy support.

We argue that objective energy cost exposure (higher energy burden) reduces households’ financial slack and increases day-to-day budget pressure. This material constraint is experienced as lower subjective economic well-being, i.e., feeling that one manages poorly financially. Lower subjective economic well-being, in turn, increases support for policies that are perceived as alleviating affordability constraints during the low-carbon transition (e.g., housing benefits). Therefore, part of the association between objective energy poverty and policy support is expected to operate indirectly through subjective economic well-being. This mechanism does not assume that all objectively energy-poor households report low subjective well-being, nor that subjective well-being fully explains policy preferences. Rather, we expect subjective economic well-being to capture one indirect pathway linking objective energy cost exposure to policy support. Empirically, this implies that the association between objective energy burden and policy support should attenuate once subjective economic well-being is included in the model.

The control variables in our approach describe respondent’s sociodemographic and housing characteristics. These variables are included in the regression analyses to account for potential confounding effects, allowing us to better isolate the relationship between our main independent variables and the dependent variable.

For the analyses, 184 respondents were excluded due to (i) missing information (e.g., household size, income, or some crucial building characteristics) to calculate the housing-related energy costs and the resulting objective energy poverty indicators, or (ii) missing or do not know responses to the dependent variable statement. The final sample included 1264 fully complete observations.

The dependent variable and its response shares are presented in Table 1. In addition, Table 2 describes dependent and independent variables for the final sample. The independent variables are classified into sociodemographic characteristics, home characteristics, and energy poverty indicators.

**Table 1:** Dependent variable

Dependent variable: society should financially support citizens in their transition to low-carbon housing	Full sample proportion % (*N* = 1448)	Final sample proportion % (*N* = 1264)
Totally disagree	4.97	5.22
Somewhat disagree	6.42	7.04
Neither agree nor disagree	17.13	18.59
Somewhat agree	42.61	44.86
Totally agree	23.14	24.29
Do not know	5.73	0.00

**Table 2:** Variable descriptions and descriptive statistics for the final sample (N = 1264). *Variable is based on a Likert scale item with 0 = totally or somewhat disagree, 1 = neither agree nor disagree, 2 = somewhat agree, and 3 = totally agree

Variables	Description	Mean	SD
*Dependent variable*			
Support*	Response to claim “I think that society should financially support citizens in their transition to low-carbon housing”	1.81	0.94
*Sociodemographic characteristics*			
Age	Respondent’s age	52.89	17.11
High education (1 if yes)	Respondent has an applied sciences or university degree	0.46	
Income (12 categories)	Household’s monthly gross income (1 = < 1000€, 2 = 1000€–1999€, 3 = 2000€-2999€, 4 = 3000–3999€, 5 = 4000–4999€, 6 = 5000–5999, 7 = 6000–6999€, 8 = 7000–7999€, 9 = 8000–8999€, 10 = 9000–9999€, 11 = 10 000–10 999€, 12 = > 11 000€)	5.40	2.89
Female (1 if yes)	Respondent is female	0.53	
Household size	Number of individuals in the household	2.31	1.25
*Home characteristics*			
Citylike environment (1 if yes)	Respondent lives in the city or municipality center	0.73	
Rental home (1 if yes)	Respondent lives in a rental home	0.21	
Detached house (1 if yes)	Respondent lives in a detached house	0.52	
Floor area	Heated floor area of respondent’s home	108.2	60.1
Electric heating (1 if yes)	Respondent’s home has direct or central electric heating	0.27	
District heating (1 if yes)	Respondent’s home has district heating	0.40	
Oil heating (1 if yes)	Respondent’s home has oil heating	0.04	
Wood heating (1 if yes)	Respondent’s home has wood heating	0.09	
*Objective energy poverty indicators*			
Energy burden	Estimated housing-related energy costs divided by household income	0.065	0.054
Energy poverty 10% (1 if yes)	Over 10% of household income is spent on housing-related energy costs	0.165	
*Subjective energy poverty indicator*			
Ordinal subjective economic well-being	Response to “How well do you think your household is managing financially today?” (0 = Poorly, 1 = Quite poorly, 2 = Moderately/Do not know, 3 = Well, 4 = Excellently)	2.61	0.87
Subjective economic well-being (1 if yes)	Respondent manages financially well or excellently	0.584	

To test our hypotheses on respondents’ answers on dependent variable, i.e., the claim “I think that society should financially support citizens in their transition to low-carbon housing,” an ordered logit regression was conducted. The responses were measured on a 5-point Likert scale. Our choice of ordered logit is motivated by the ordinal nature of the dependent variable and the need to model the underlying latent structure appropriately. The ordered logit regression considers nonlinearities in the survey responses and treats the responses as rankings (Greene 2018). To satisfy the proportional odds assumption of the ordered logit regression model, two response categories with relatively few observations were combined (totally disagree and somewhat disagree; see Table 1).

Here, $y_{i}$ is observed indicating the response of the individual *i* on the statement. More formally, we can define:

1 $$\left\{ {\begin{array}{*{20}l} {y_{i} = 0 \left( {{\mathrm{totally}}\,\,{\mathrm{or}}\,\,{\mathrm{somawhat}}\,\,{\mathrm{disagree}}} \right) \,{\mathrm{if}}\,\,y_{i}^{*} \le \mu_{0} } \hfill \\ {y_{i} = 1 \left( {{\mathrm{neither}}\,\,{\text{agree }}\,\,{\text{nor }}\,\,{\mathrm{disagree}}} \right)\,\,{\text{ if}}\,\,\mu_{0} < y_{i}^{*} \le \mu_{1} } \hfill \\ {y_{i} = 2 \left( {{\mathrm{somewhat}}\,\,{\mathrm{agree}}} \right)\,\,{\mathrm{if}}\,\, \mu_{1} < y_{i}^{*} \le \mu_{2} } \hfill \\ {y_{i} = 3 \left( {{\mathrm{totally}}\,\,{\mathrm{agree}}} \right) \,\,{\mathrm{if}}\,\, y_{i}^{*} > \mu_{2} } \hfill \\ \end{array} } \right.,$$

where $y_{i}^{*}$ is the unobserved latent variable related to the response of the individual *i* and $\mu_{0}$ to $\mu_{2}$ correspond to the threshold (i.e., cutoff) parameters. Further, the regression model can be written as:

2 $$\begin{array}{*{20}c} {y_{i}^{*} = \beta^{\prime } X_{i} + \varepsilon_{i} ,} \\ \end{array}$$

where $X_{i}$ is the vector of independent variables, β are the corresponding coefficients, and $\varepsilon_{i}$ is the error term. The coefficients are estimated by maximum likelihood method. The analyses were done using NLOGIT and R.

## Results

Overall, the vast majority of the respondents either somewhat agreed (45%) or totally agreed (24%) that society should financially support citizens in their transition to low-carbon housing (Table 1). Only around 12.3% of respondents totally or somewhat disagreed with this idea. Regarding energy poverty indicators (Table 2), 16.5% of the respondents used over 10% of their household income on housing-related energy costs. Moreover, respondents indicated to manage, on average, moderately to well financially (see variable *Subjective economic well-being*). As expected, the objective energy poverty indicators *Energy burden* and *Energy poverty 10%* and proxy for subjective energy poverty, i.e., *Subjective economic well-being*, are negatively correlated. The Pearson correlation between *Energy poverty 10%* and *Subjective economic well-being* is − 0.188, whereas the Pearson correlation between *Energy burden* and *Subjective economic well-being* is − 0.244. Of those who stated that they manage either poorly, quite poorly, or moderately financially today, approximately 23.8% were using over 10% of their income on housing-related energy costs. Among respondents who reported managing financially poorly or moderately, those below the 10% threshold are more likely to live in urban rental housing and apartment buildings and to use district heating (e.g., district heating 47.4% vs. 11.2%, detached houses 40.1% vs. 75.2%). This pattern helps to explain why some financially strained households do not exceed the 10% threshold: their energy costs are relatively lower, while their reported financial difficulties likely reflect other budget pressures.

Tables 3 and 4 present the results of the ordered logit regressions. In both tables, the five models incorporate different sets of independent variables, following the classification outlined in Table 2. To assess potential multicollinearity, we examined correlations and variance inflation factors (VIFs) among the explanatory variables and found no evidence of multicollinearity affecting the results (see Supplementary Information, Appendix S2 Tables S1 and S2). All VIFs were below 2, and the largest absolute correlation between explanatory variables was 0.41. Interpreting coefficients in ordered logit models is not straightforward; however, the signs indicate the direction of each variable’s influence on respondents' levels of agreement with the support statement, as captured by the latent dependent variable. Across all models, several variables significantly influence the probability distribution of the latent outcome. While cutoff parameters are essential for model estimation, they are not meaningful for interpretation.

**Table 3:** Results of the ordered logit regression for energy burden. Standard errors are shown in the parentheses. *, **, and *** indicate significance at the 0.1, 0.05, and 0.01 levels, respectively. AIC: Akaike information criterion. Reference groups are *Manages poorly, quite poorly, or moderately financially*; *Low education; Male; Rural environment; Owned home; and Apartment buildings and row houses*

	Model 1	Model 2	Model 3	Model 4	Model 5
Energy burden	1.665* (0.954)	0.862 (0.975)	1.528 (1.057)	2.893** (1.161)	2.648** (1.165)
Subjective economic well-being		− 0.437*** (0.109)			− 0.434*** (0.119)
Income			− 0.030 (0.022)	− 0.014 (0.023)	0.013 (0.024)
Age			− 0.007** (0.003)	− 0.002 (0.004)	− 0.003 (0.004)
High education			0.211* (0.111)	0.165 (0.113)	0.199* (0.113)
Female			0.382*** (0.105)	0.387*** (0.105)	0.397*** (0.105)
Household size			− 0.047 (0.046)	− 0.007 (0.049)	− 0.035 (0.049)
Citylike environment				0.310** (0.135)	0.303** (0.135)
Rental home				0.299* (0.164)	0.240 (0.165)
Detached house				− 0.096 (0.135)	− 0.086 (0.135)
Cutoff 1|2	− 1.864*** (0.104)	− 2.190*** (0.133)	− 2.232*** (0.264)	− 1.528*** (0.341)	− 1.745*** (0.347)
Cutoff 2|3	− 0.706*** (0.085)	− 1.027*** (0.118)	− 1.058*** (0.257)	− 0.345 (0.337)	− 0.558 (0.342)
Cutoff 3|4	1.250*** (0.091)	0.950*** (0.117)	0.929*** (0.256)	1.657*** (0.340)	1.462*** (0.344)
Log likelihood	− 1606.6	− 1598.5	− 1592.9	− 1586.2	− 1579.5
Log likelihood (constants only)	− 1608.2	− 1608.2	− 1608.2	− 1608.2	− 1608.2
AIC	3221.3	3207.1	3203.8	3196.4	3185.1
N	1264	1264	1264	1264	1264

**Table 4:** Results of the ordered logit regression for energy poverty share of 10%. Standard errors are shown in the parentheses. *, **, and *** indicate significance at the 0.1, 0.05, and 0.01 levels, respectively. AIC: Akaike information criterion. Reference groups are *Energy burden less than 10%*; *Manages poorly, quite poorly, or moderately financially*; *Low education; Male; Rural environment; Owned home; and Apartment buildings and row houses*

	Model 1	Model 2	Model 3	Model 4	Model 5
Energy poverty 10%	0.312** (0.142)	0.213 (0.145)	0.290* (0.152)	0.428*** (0.160)	0.396** (0.160)
Subjective economic well-being		− 0.429*** (0.108)			− 0.433*** (0.119)
Income			− 0.030 (0.021)	− 0.019 (0.022)	0.008 (0.023)
Age			− 0.007** (0.003)	− 0.003 (0.004)	− 0.003 (0.004)
High education			0.212* (0.111)	0.167 (0.113)	0.201* (0.113)
Female			0.375*** (0.105)	0.378*** (0.105)	0.389*** (0.105)
Household size			− 0.045 (0.046)	− 0.003 (0.049)	− 0.032 (0.049)
Citylike environment				0.297** (0.134)	0.291** (0.134)
Rental home				0.306* (0.164)	0.246 (0.165)
Detached house				− 0.072 (0.132)	− 0.064 (0.132)
Cutoff 1|2	− 1.924*** (0.088)	− 2.208*** (0.115)	− 2.295*** (0.254)	− 1.677*** (0.325)	− 1.880*** (0.331)
Cutoff 2|3	− 0.765*** (0.064)	− 1.045*** (0.097)	− 1.121*** (0.246)	− 0.494 (0.320)	− 0.693** (0.325)
Cutoff 3|4	1.194*** (0.070)	0.934*** (0.095)	0.869*** (0.245)	1.509*** (0.323)	1.328*** (0.327)
Log likelihood	− 1605.8	− 1597.8	− 1592.1	− 1585.7	− 1579.1
Log likelihood (constants only)	− 1608.2	− 1608.2	− 1608.2	− 1608.2	− 1608.2
AIC	3219.5	3205.7	3202.2	3195.5	3184.2
N	1264	1264	1264	1264	1264

Regarding the analysis for *Energy burden* (Table 3), we found that those respondents with a higher level of energy burden were more likely to prefer public support for transition to low-carbon housing, which is in line with our expectations (H1). Controlling for sociodemographic background variables (Model 3) somewhat attenuated the coefficient, while adding the remaining control variables (Model 4) clearly amplified it.

Quite logically and following our expectations, those who felt that they were doing well economically (variable *Subjective economic well-being*), were more reluctant for financial support. In addition, controlling for this subjective measure does partly explain why objective energy burden is related to this policy preference. Thus, when comparing Models 1 and 2, H2 was supported by our analysis: The association of objective energy poverty and policy support for transition to low-carbon housing is explained by weakened subjective economic well-being. In other words, the statistical significance of energy burden disappears when taking subjective economic well-being into consideration. However, support for the hypothesis is not as evident when control variables are included in the model. To further assess this mechanism, we also estimated logit models in Appendix S2 (Table S1), where subjective economic well-being was regressed on objective energy burden and the control variables.

In the Models 3–5, we see also that women (*Female*), higher educated (*High education*), and urban dwellers (*Citylike environment*) are more supportive for public funding. Running the ordered logit analyses with the binary *Energy poverty 10%* indicator essentially replicates all the above-mentioned results (see Table 4). As a robustness check, we also estimated the Model 5 in Table 3 and Model 5 in Table 4 using a more granular seven-category urban–rural classification (see Table S4 in Appendix S2). The results were consistent with those based on the aggregated residence indicator, in both direction and magnitude, with higher support for low-carbon policies observed in regional centers relative to rural areas.

We also estimated models including home characteristics which were used in the calculation of the objective energy poverty variables. These were heating systems and floor area information. The results of these full models are presented in Supplementary Information in Appendix S2 Table S5, and the findings generally are in line with the main models presented here. Home characteristics capture the relationship related to the objective energy poverty indicators, but the subjective economic well-being measure still remains statistically significant. The results show that *Oil heating* is associated with higher likelihood to prefer public funding for low-carbon housing. Oil-heated households in our sample differ systematically from others, which helps interpret their stronger support for public funding. Their average energy burden is markedly higher (13.3% vs. 6.2%), and they are more likely to live in rural areas (52.2% vs. 26.1%) and in owner-occupied detached houses, suggesting a more rural, higher-exposure subgroup.

Lastly, we conducted a sensitivity analysis in which we replaced the 2022 electricity energy and heating oil prices with 2021 average prices (electric energy 6.33 cents/kWh, heating oil 10.1 cents/kWh), and assumed that all wood fuel is received for free from own forests. Under these assumptions, the share of households classified as energy poor using the 10% threshold decreases from 16.5 to 8.0%. We also re-estimated the regression models, and the results are reported in Tables S6 and S7 in Appendix S2. Overall, the findings are consistent with the main results in Tables 3 and 4, particularly with respect to the direction and statistical significance of the key variables (*Energy burden*, *Energy poverty 10%,* and *Subjective economic well-being*).

## Discussion and conclusion

Our findings indicated strong public endorsement for the notion that society should financially support citizens in transitioning to low-carbon housing. Finnish government thus maintains a strong public push to continue enabling decarbonization in the residential housing. This finding can be compared with earlier studies such as Odland et al. (2023), who found that a clear majority of Canadian homeowners support subsidy-based policies (e.g., grants and low-interest loans) for housing decarbonization, while showing significantly less support for compulsory measures like carbon taxes or regulatory mandates. Similarly, Rhodes et al. (2017) reported high levels of public support for voluntary climate policy instruments, including subsidies for energy-efficient technologies, whereas support for carbon taxes was markedly lower.

Regarding energy poverty, we hypothesized that objective energy poverty would be associated with stronger preference for public-funded low-carbon housing policy (H1) and that this association would run partly via subjective economic well-being (H2). Our findings supported H1 with two different objective energy poverty indicators. In addition, the mediation hypothesis H2 gained some support. The findings suggest that objective energy poverty is linked to general subjective economic well-being, which is further associated with preferences for decarbonization policies.

The finding that energy poverty is associated with stronger support for low-carbon housing policies contrasts with some earlier literature suggesting that energy-poor households may be less supportive of low-carbon transition policies due to affordability concerns (Chapman and Okushima 2019). However, this apparent discrepancy may stem from differences in policy design, framing, and expected distributional outcomes. In particular, Chapman and Okushima (2019) focused on public support for solar panel feed-in tariffs, a policy from which energy-poor households were largely excluded and for which benefits were not equitably distributed. By contrast, many existing studies indicate that energy-poor and vulnerable households demonstrate a willingness to engage in decarbonization efforts, if inequities in financial opportunities and limitations in participatory voice are adequately addressed (Tozer et al. 2023; Irwin 2025).

The share of objectively energy-poor households, using over 10% of income on housing-related energy costs, was 16.5% in our data reflecting high energy prices during that time. This seems to be in line with findings of other Finnish study from year 2022 by Numminen et al. (2024). In Finland, part of housing-related energy expenditure, particularly heating in row houses and apartment buildings, is often embedded in general maintenance charges or rents. However, our modeling approach captures these “hidden” energy costs, which may lead to higher estimated energy burdens than approaches based solely on directly reported household energy bills. Moreover, Maier and Dreoni (2026) reported that approximately 17% of the population also on the EU level is classified as energy poor according to expenditure-based indicators which is nearly double the 8–9% observed subjective measures. Furthermore, Numminen et al. (2024) suggested that objective energy poverty is overlapping with subjective energy poverty for 8% of households in Finland. This holds also in our study as we found moderate negative correlations between our objective energy poverty indicators and subjective economic well-being. In our sample, less respondents were objectively energy poor than those who stated poor or moderate economic well-being (16.5% vs. 41.6%). This demonstrates that objective energy poverty indicators capture a narrower, energy-specific constraint, whereas self-reported economic well-being reflects broader financial stress.

Moreover, the objective energy poverty indicators used in this study are based on proportional shares. Thus, they do not account for the fact that the absolute amount of remaining income available for other expenditures increases with income. In other words, even when energy costs constitute at least 10% of total expenditure, the residual income available for other purposes is substantially higher for households earning, for example, 10 000€ per month compared to those earning 2000€ per month, implying very different levels of financial constraint despite identical indicator values. Conversely, some households may report financial strain even below the 10% threshold if other necessary expenditures are high, while some high-income households may exceed 10% without experiencing comparable hardship. This highlights why subjective economic well-being is a relevant complementary measure as it reflects perceived financial slack after meeting all necessary expenses, and may therefore better capture how a given objective energy burden translates into experienced economic strain and, ultimately, policy preferences.

Our study also revealed that individuals who were female, had a high education degree**,** lived in urban areas, and used oil heating were more likely to favor financial support from society to help citizens transition to low-carbon housing. Previous research has also found a link between climate policy subsidies and demographic and contextual characteristics (Douenne and Fabre 2020; Spandagos et al. 2022; Odland et al. 2023). Rhodes et al. (2017) found that women were generally more supportive of climate policies, especially non-coercive measures such as subsidies. Our finding regarding urban areas aligns with Douenne and Fabre (2020) and Spandagos et al. (2022) who found that urban residents are more supportive of climate policies.

Moreover, the higher level of support observed among respondents using oil-based heating likely reflects the combination of its high carbon intensity and substantial financial burden. For these households, public support may be particularly salient in enabling a transition to cleaner heating systems. This interpretation is reinforced by recent evidence from Numminen et al. (2024), who showed that Finnish households relying on oil heating are more likely to become energy vulnerable.

It is important to recognize the heterogeneity among households. In Finland, higher-income households are eligible to apply for subsidies, despite often facing less urgent need than lower-income households. For example, they have benefited disproportionately from subsidies supporting the phaseout of oil heating. Our findings showed higher policy support among energy-poor households and were consistent with earlier research documenting a negative relationship between income and energy poverty (del Río et al. 2025). Taken together, these results indirectly suggest that sociodemographic factors, particularly income, should be more carefully considered in the design and targeting of subsidy schemes (see also Schleich (2019)). Since reliable measurement of energy poverty and related database still require development, the risk of energy poverty could be assessed based on the characteristics of residential building and household (including household income) obtained from registers. Financial support should be directed specifically at those at risk of energy poverty. This approach would ensure that those who genuinely need financial assistance receive it, thereby enhancing the overall effectiveness of the support system.

Although our study provides interesting insights, it is not without limitations. Regarding the calculation of energy poverty indicators, citizens’ energy consumption was modeled rather than directly observed and energy price estimates were primarily based on national average values, which may introduce some inaccuracies. Moreover, citizen perceptions of low-carbon housing policy were measured with one general policy statement in this study. In turn, the analyses add understanding only on factors associated with support for general subsidy type of policies. Additionally, while the model identifies associations between energy burden, demographics, and policy attitudes, it does not capture dynamic or context-dependent factors, such as over time varying energy prices or evolving public discourse on climate change mitigation and adaptation measures in Finland. Moreover, omitted variables, such as individual experiences with energy retrofits or political leaning, may influence responses, raising the risk of unobserved confounding.

The timing of this study should be considered when interpreting the results. In Finland, consumer energy prices increased by 31% in 2022, primarily due to the Russian invasion of Ukraine and the cessation of electricity imports from Russia (State Treasury 2023). For example, the surges in electricity prices affected Finnish households unevenly: some retained older, cheaper fixed term electricity contracts, while others paid spot market prices. Overall, the year 2022 was exceptional in terms of energy prices, which may influence the results in two ways. First, the share of households objectively classified as energy poor was higher in 2022–2023 than in previous years. However, the regression models regarding the hypotheses remained robust when the objective energy poverty indicators were calculated using 2021 prices. Thus, in this respect, the results do not appear to be strongly linked to the year of data collection. Second, the situation may have shaped respondents’ survey answers: their attitudes toward financial support for the transition and their perceived subjective economic well-being. However, the energy crisis peaked later in 2022, after the survey was conducted, so its impact on responses is likely limited.

These limitations need to be kept in mind when interpreting the findings. Thus, future research could investigate a broader set of low-carbon housing policies, compare citizen perceptions for such policies across EU countries and over time, and collect even a richer set of background information. Ideally, panel data would help in addressing the issue of unobserved heterogeneity.

Overall, our findings contribute to the emerging body of literature that connects housing affordability, energy poverty, and climate policy acceptance, highlighting the importance of addressing social vulnerabilities to ensure broad support for and just transition to climate-wise housing.

## Supplementary Information

Below is the link to the electronic supplementary material.

Supplementary file1 (PDF 501 KB)

## Data Availability

Data protection statement provided to respondents prohibits publicly disclosing data.
