# Supplementary material for: How is energy poverty linked with citizen perceptions of financial support for low-carbon housing?
Source: Ambio. 2026 Mar 17;55(9):2094–106. doi: 10.1007/s13280-026-02364-4 (PMC13438078; doi:10.1007/s13280-026-02364-4)
Supplement: Supplementary file 1 [file 13280_2026_2364_MOESM1_ESM.pdf]

Supplementary Information for "How is energy poverty linked with citizen perceptions of financial support for low-carbon housing?"

This Supplementary Information has not been peer reviewed.

It includes Survey questions (Appendix S1) and Additional results (Appendix S2).

Appendix S1

Survey questions used in this study

What type of building do you currently live in?
- Block of flats
- Detached house
- Semi-detached house
- Terraced house
- Other, what? ___________________________

What kind of area do you currently live in?
- City centre
- Urban residential area with a majority of blocks of flats
- Urban residential area with a majority of single-family houses
- Local centre in the countryside
- Sparsely populated area in the countryside
- Other, what? ______________________________________

What is the size of your home in square meters? (In detached and single-family houses count only the heated area.)

______ Square meters.

What is the type of ownership of your housing?
- Owner-occupied dwelling
- Rental dwelling
- Right-of-occupancy dwelling
- Other, what? ________________________________

What is the primary form of heating in your home?
- District heat
- Direct electric heating
- Water cycle electric heating
- Geothermal heating
- Air-to-water heat pump
- Exhaust air heat pump
- Wood heating (e.g. wood or wood chips)
- Pellet heating
- Oil heating
- Other, what?
- I cannot say

Does your home have some of the following? Select all appropriate options.
- Solar thermal collector
- Photovoltaic panels
- Air source heat pump
- Heat-storing fireplace

- Wood-burning oven
- Convection fireplace
- Water-circulating fireplace
- Other type of fireplace
- Electric radiator or underfloor heating for additional heating
- None of these
- I cannot say

Do you agree or disagree with the following statements on the carbon footprint of housing?
Response options: strongly disagree, somewhat disagree, neither agree nor disagree, somewhat agree, strongly agree, I cannot say

a) I think that the carbon footprint of housing is too big in Finland.
b) I think that there is enough information available on low-carbon housing.
c) I think that society should financially support citizens in their transition to low-carbon housing.
d) I believe that the value of houses will increase when their carbon footprints are reduced.
e) I think that renovating and repairing buildings should be favoured instead of tearing them down and building new ones.
f) I think it is important to use wood as the main material in new residential buildings.
g) I feel that low-carbon housing is possible for anyone in Finland.

Gender

- Woman
- Man
- Other
- I wish not to tell

Year of birth (Enter in the form yyyy, e.g., 1980) _______________

Level of education (Select your highest level of education.)

- Primary or elementary school
- Vocational upper secondary qualification and/or matriculation examination/high school
- Further vocational training
- Bachelor's degree or equivalent
- Master's degree or equivalent
- Licenciate or doctoral degree
- Other, what?

How much is the combined monthly gross income of your household?

- Less than 1 000 €
- 1 000–1 999 €
- 2 000–2 999 €
- 3 000–3 999 €
- 4 000–4 999 €
- 5 000–5 999 €
- 6 000–6 999 €
- 7 000–7 999 €
- 8 000–8 999 €

- 9 000–9 999 €
- 10 000–10 999 €
- More than 11 000 €

How well do you think your household is managing financially today?

- Poorly
- Quite poorly
- Moderately
- Well
- Excellently
- I cannot say

Appendix S2

Table S1. Correlation matrix.

	Energy burden	Energy poverty 10%	Income	Age	High education	Female	Household size	Citylike environment	Rental home	Detached house	Subjective economic wellbeing
Energy burden	1.000	0.760	-0.378	0.072	-0.187	0.013	-0.013	-0.311	-0.070	0.313	-0.244
Energy poverty 10%		1.000	-0.307	0.094	-0.163	0.041	-0.038	-0.251	-0.080	0.247	-0.188
Income			1.000	0.020	0.283	-0.050	0.326	0.118	-0.306	0.169	0.459
Age				1.000	-0.197	0.012	-0.226	-0.127	-0.408	0.187	0.048
High education					1.000	-0.010	0.087	0.184	0.031	-0.132	0.212
Female						1.000	-0.053	0.009	-0.011	-0.047	0.023
Household size							1.000	-0.056	-0.174	0.287	0.000
Citylike environment								1.000	0.179	-0.409	0.076
Rental home									1.000	-0.450	-0.217
Detached house										1.000	0.039
Subjective economic wellbeing											1.000

Table S2. Variance inflation factors.

	Energy burden	Energy poverty 10%
Energy burden	1.450	
Energy poverty 10%		1.247
Income	1.820	1.702
Age	1.391	1.392
High education	1.176	1.176
Female	1.012	1.012
Household size	1.380	1.379
Citylike environment	1.303	1.287
Rental home	1.590	1.590
Detached house	1.681	1.613
Subjective economic wellbeing	1.244	1.243

Table S3. Logit regression models explaining subjective economic wellbeing.

	Model 1	Model 2	Model 3	Model 4
Constant	0.877***<br>(0.100)	-0.421<br>(0.418)	0.489***<br>(0.063)	-0.519<br>(0.395)
Energy burden	-8.347***<br>(1.278)	-1.823<br>(1.474)		
Energy poverty 10%			-0.887***<br>(0.154)	-0.251<br>(0.187)
Income		0.361***<br>(0.035)		0.364***<br>(0.034)
Age		-0.005<br>(0.004)		-0.005<br>(0.004)
High education		0.418***<br>(0.139)		0.417***<br>(0.140)
Female		0.098<br>(0.130)		0.103<br>(0.130)
Household size		-0.382***<br>(0.067)		-0.384***<br>(0.067)
Citylike environment		0.041<br>(0.161)		0.049<br>(0.161)
Rental home		-0.708***<br>(0.198)		-0.710***<br>(0.198)
Detached house		0.136<br>(0.174)		0.122<br>(0.170)
Log likelihood	-832.9	-710.4	-841.5	-710.3
AIC	1669.7	1440.9	1686.9	1440.7
N	1264	1264	1264	1264

Note: Standard errors are shown in the parentheses. *, **, and *** indicate significance at the 0.1, 0.05, and 0.01 levels, respectively. AIC: Akaike Information Criterion. Reference groups are *Energy burden less than 10%*; *Manages poorly, quite poorly, or moderately financially*; *Low education; Male, Rural environment; Owned home; and Apartment buildings and row houses.*

Table S4. Ordered logit regression results with different regional classification.

	Model 1	Model 2	Model 3	Model 4
Energy burden	2.648**<br>(1.165)	2.692**<br>(1.175)		
Energy poverty 10%			0.396**<br>(0.160)	0.409**<br>(0.162)
Subjective economic wellbeing	-0.434***<br>(0.119)	-0.448***<br>(0.120)	-0.433***<br>(0.119)	-0.447***<br>(0.120)
Income	0.013<br>(0.024)	0.013<br>(0.024)	0.008<br>(0.023)	0.009<br>(0.023)
Age	-0.003<br>(0.004)	-0.003<br>(0.004)	-0.003<br>(0.004)	-0.003<br>(0.004)
High education	0.199*<br>(0.113)	0.185<br>(0.115)	0.201*<br>(0.113)	0.186<br>(0.115)
Female	0.397***<br>(0.105)	0.393***<br>(0.105)	0.389***<br>(0.105)	0.385***<br>(0.105)
Household size	-0.035<br>(0.049)	-0.034<br>(0.049)	-0.032<br>(0.049)	-0.031<br>(0.049)
Citylike environment	0.303**<br>(0.135)		0.291**<br>(0.134)	
Inner urban area		0.457**<br>(0.221)		0.455**<br>(0.221)
Outer urban area		0.366*<br>(0.217)		0.358*<br>(0.216)
Rural local centers		0.520*<br>(0.301)		0.527*<br>(0.301)
Peri-urban area		0.351<br>(0.246)		0.336<br>(0.246)
Rural area close to a city		0.129<br>(0.258)		0.138<br>(0.258)
Sparsely populated rural area		-0.258<br>(0.291)		-0.258<br>(0.291)
Rental home	0.240<br>(0.165)	0.244<br>(0.166)	0.246<br>(0.165)	0.250<br>(0.166)
Detached house	-0.086<br>(0.135)	-0.030<br>(0.148)	-0.064<br>(0.132)	-0.005<br>(0.156)
Cutoff 1|2	-1.745***<br>(0.347)	-1.640***<br>(0.384)	-1.880***<br>(0.331)	-1.769***<br>(0.367)
Cutoff 2|3	-0.558<br>(0.342)	-0.449<br>(0.380)	-0.693**<br>(0.325)	-0.577<br>(0.363)
Cutoff 3|4	1.462***<br>(0.344)	1.578***<br>(0.382)	1.328***<br>(0.327)	1.452***<br>(0.364)
Log likelihood	-1579.5	-1576.5	-1579.1	-1575.9
Log likelihood (constants only)	-1608.2	-1608.2	-1608.2	-1608.2
AIC	3185.1	3189.0	3184.2	3187.9
N	1264	1264	1264	1264

Note: Standard errors are shown in the parentheses. *, **, and *** indicate significance at the 0.1, 0.05, and 0.01 levels, respectively. AIC: Akaike Information Criterion. Reference groups are *Energy burden less than 10%*; *Manages poorly, quite poorly, or moderately financially*; *Low education; Male; Core rural area; Owned home; and Apartment buildings and row houses.*

Table S5. Results of the ordered logit regressions with floor area and heating methods.

	Model 1	Model 2
Energy burden	2.072<br>(1.439)	
Energy poverty 10%		0.285<br>(0.178)
Subjective economic wellbeing	-0.447***<br>(0.120)	-0.448***<br>(0.119)
Income	0.011<br>(0.026)	0.005<br>(0.024)
Age	-0.003<br>(0.004)	-0.004<br>(0.004)
High education	0.210*<br>(0.114)	0.207*<br>(0.114)
Female	0.392***<br>(0.106)	0.388***<br>(0.106)
Household size	-0.035<br>(0.051)	-0.036<br>(0.051)
Citylike environment	0.291**<br>(0.140)	0.277**<br>(0.140)
Rental home	0.235<br>(0.167)	0.246<br>(0.167)
Detached house	-0.034<br>(0.176)	-0.041<br>(0.176)
Floor area	0.000<br>(0.002)	0.000<br>(0.001)
Electric heating	0.179<br>(0.158)	0.196<br>(0.156)
District heating	0.198<br>(0.163)	0.205<br>(0.164)
Oil heating	0.986***<br>(0.335)	0.970***<br>(0.335)
Wood heating	0.040<br>(0.231)	0.030<br>(0.231)
Cutoff 1|2	-1.667***<br>(0.366)	-1.766***<br>(0.350)
Cutoff 2|3	-0.476***<br>(0.361)	-0.575*<br>(0.345)
Cutoff 3|4	1.558***<br>(0.364)	1.460***<br>(0.347)
Log likelihood	-1574.4	-1574.2
Log likelihood (constants only)	-1608.2	-1608.2
AIC	3184.9	3184.4
N	1264	1264

Note: Standard errors are shown in the parentheses. *, **, and *** indicate significance at the 0.1, 0.05, and 0.01 levels, respectively. AIC: Akaike Information Criterion. Reference groups are *Energy burden less than 10%*; *Manages poorly, quite poorly, or moderately financially*; *Low education; Male; Rural environment; Owned home; Apartment buildings and row houses; and Other heating*.

Table S6. Results of the ordered logit regression for energy burden with energy prices in 2021.

	Model 1	Model 2	Model 3	Model 4	Model 5
Energy burden	2.720** (1.312)	1.659 (1.335)	2.322 (1.442)	3.067** (1.487)	2.824* (1.165)
Subjective economic wellbeing		-0.429*** (0.109)			-0.440*** (0.119)
Income			-0.028 (0.021)	-0.020 (0.023)	0.009 (0.024)
Age			-0.007** (0.003)	-0.003 (0.004)	-0.003 (0.004)
High education			0.208* (0.111)	0.165 (0.113)	0.200* (0.113)
Female			0.380*** (0.105)	0.386*** (0.105)	0.396*** (0.105)
Household size			-0.045 (0.046)	-0.007 (0.049)	-0.035 (0.049)
Citylike environment				0.269** (0.134)	0.265** (0.134)
Rental home				0.291* (0.164)	0.231 (0.165)
Detached house				-0.053 (0.132)	-0.047 (0.132)
Cutoff 1|2	-1.842*** (0.105)	-2.162*** (0.133)	-2.205*** (0.267)	-1.611*** (0.336)	-1.822*** (0.341)
Cutoff 2|3	-0.683*** (0.086)	-0.999*** (0.118)	-1.031*** (0.260)	-0.429 (0.331)	-0.636* (0.336)
Cutoff 3|4	1.274*** (0.092)	0.979*** (0.118)	0.956*** (0.259)	1.570*** (0.334)	1.382*** (0.338)
Log likelihood	-1606.0	-1598.2	-1592.6	-1587.2	-1580.3
Log likelihood (constants only)	-1608.2	-1608.2	-1608.2	-1608.2	-1608.2
AIC	3220.0	3206.3	3203.3	3198.4	3186.7
N	1264	1264	1264	1264	1264

Note: Standard errors are shown in the parentheses. *, **, and *** indicate significance at the 0.1, 0.05, and 0.01 levels, respectively. AIC: Akaike Information Criterion. Reference groups are *Manages poorly, quite poorly, or moderately financially*; *Low education; Male; Rural environment; Owned home; and Apartment buildings and row houses.*

Table S7. Results of the ordered logit regression for energy poverty share of 10% with energy prices in 2021.

	Model 1	Model 2	Model 3	Model 4	Model 5
Energy poverty 10%	0.418**<br>(0.194)	0.318<br>(0.196)	0.325<br>(0.203)	0.399*<br>(0.206)	0.386**<br>(0.206)
Subjective economic wellbeing		-0.434***<br>(0.107)			-0.446***<br>(0.119)
Income			-0.034<br>(0.021)	-0.027<br>(0.022)	0.002<br>(0.023)
Age			-0.007**<br>(0.003)	-0.002<br>(0.004)	-0.003<br>(0.004)
High education			0.208*<br>(0.111)	0.168<br>(0.113)	0.202<br>(0.113)
Female			0.372***<br>(0.105)	0.377***<br>(0.105)	0.387***<br>(0.105)
Household size			-0.040<br>(0.046)	-0.003<br>(0.049)	-0.032<br>(0.049)
Citylike environment				0.273**<br>(0.134)	0.268**<br>(0.134)
Rental home				0.292*<br>(0.164)	0.231<br>(0.165)
Detached house				-0.028<br>(0.130)	-0.025<br>(0.130)
Cutoff 1|2	-1.940***<br>(0.087)	-2.221***<br>(0.112)	-2.299***<br>(0.254)	-1.738***<br>(0.323)	-1.940***<br>(0.328)
Cutoff 2|3	-0.780***<br>(0.062)	-1.057***<br>(0.094)	-1.124***<br>(0.246)	-0.557*<br>(0.318)	-0.753**<br>(0.323)
Cutoff 3|4	1.177***<br>(0.068)	0.922***<br>(0.092)	0.863***<br>(0.245)	1.441***<br>(0.320)	1.264***<br>(0.324)
Log likelihood	-1605.8	-1597.6	-1592.7	-1587.4	-1580.4
Log likelihood (constants only)	-1608.2	-1608.2	-1608.2	-1608.2	-1608.2
AIC	3219.7	3205.2	3202.3	3198.9	3186.8
N	1264	1264	1264	1264	1264

Note: Standard errors are shown in the parentheses. *, **, and *** indicate significance at the 0.1, 0.05, and 0.01 levels, respectively. AIC: Akaike Information Criterion. Reference groups are *Energy burden less than 10%*; *Manages poorly, quite poorly, or moderately financially*; *Low education; Male; Rural environment; Owned home; and Apartment buildings and row houses.*
